# Prevalence of depression, anxiety, and associated factors in immigrant pregnant women in Türkiye: A cross-sectional study

**DOI:** 10.1097/MD.0000000000036616

**Published:** 2023-12-29

**Authors:** Muhammed Atak, Mehmet Akif Sezerol, Elif Nur Koçak, Mehmet Sait Değer, Hamza Kurubal

**Affiliations:** a Department of Public Health, Istanbul University – Istanbul Faculty of Medicine, Istanbul, Turkey; b Department of Public Health, Istanbul Medipol University – School of Medicine, Istanbul, Turkey; c Department of Public Health, Sultangazi District Health Directorate, Turkish Ministry of Health – Istanbul Health Directorate, Istanbul, Turkey; d Department of Public Health, Hitit University – Faculty of Medicine, Çorum, Turkey; e Department of Orthopedics, Porta Westfalica Clinic, Bad Oeynhausen, Germany.

**Keywords:** anxiety, depression, immigrant, mental health, pregnant

## Abstract

The prevalence of depression and anxiety in pregnant women is increasing and this is more important in migrants who are in the vulnerable group. The aim of this study was to evaluate the prevalence of depression and anxiety in Syrian immigrant pregnant women admitted to a Migrant Health Center and the associated factors. The study is a cross-sectional study. A questionnaire prepared using sociodemographic characteristics and the Beck depression and anxiety inventories in Arabic was applied to the participants. The study was conducted with immigrant pregnant women who applied to the Migrant Health Center between July 8, 2022, and December 31, 2022. The study was conducted through health workers who speak Arabic and had received training on the subject beforehand. The mean age of the research group was 26.67 ± 5.98 years. Of the participants, 73.0 percent (%) had primary education or less, 95.0% had low income, 87.2% had 3 or less children and 98.5% described their health status as good or very good. The mean Beck anxiety inventory score of the participants was found to be 4.65 ± 3.17. The mean Beck depression inventory score was found to be 4.48 ± 2.53. Smoking and first pregnancy status were found to be associated with anxiety, whereas smoking and history of delivery beyond 42 weeks were found to be associated with depression (*P* ˂ .05). Anxiety and depression levels of immigrant pregnant women were found to be very low. Minimal anxiety was found in 99.3% of the immigrant pregnant women and 97.1% had no symptoms of depression. It is thought that effective psychosocial support activities for immigrant pregnant women should be carried out in a way to cover the entire target group.

## 1. Introduction

Pregnancy is a physiological process, and some changes occur in the expectant mother during this process. However, some of the physiological changes during pregnancy may lead to undesirable outcomes.^[1]^ The causes of this condition include hormonal changes, pregnancy-related stress, social factors, and worries experienced during the preparation for pregnancy. Studies show that the frequency of depression and anxiety is significantly higher in pregnant women compared to nonpregnant women.^[2,3]^ Since migration affects the health and social status of the individual, immigrant pregnant women are at higher risk especially in terms of mental problems.^[4]^ Therefore, it is important for individual and community health to determine the health status of migrant pregnant women and to plan and implement the health services they will need during pregnancy.

Depression and anxiety are mental disorders that are frequently observed in pregnant women and may occur together.^[5]^ The prevalence of depression and depressive symptoms in pregnancy varies between 12% to 36%.^[6]^ There are many factors that cause pregnancy depression. In addition to biological and hormonal factors, environmental factors may also trigger depression in pregnant women.^[7,8]^

Immigrant pregnant women may face greater challenges both in trying to adapt to local communities and in trying to manage the pregnancy process.^[9]^ These challenges may affect the emotional health of women in this vulnerable period and increase the risk of pregnancy-related complications and poor health outcomes. Depression during pregnancy may lead to problems in the newborn, unfavorable pregnancy outcomes, and obstetric complications.^[6,10]^ Immigrant mothers may also be negatively affected by reasons such as poverty, loneliness and language problems.^[11]^ In addition, socioeconomic factors, educational level, lack of social support, and past life traumas are known to increase the risk of depression and anxiety in immigrant pregnant women.^[12]^

Antenatal care is critical for healthy pregnancy outcomes. In addition to basic needs such as shelter, nutrition and safety, migrant pregnant women should receive prenatal care that is appropriate for their specific situation. Sociocultural factors, economic status, language barriers and difficulties in accessing health services may prevent migrant pregnant women from receiving adequate prenatal care.^[13]^ Effective antenatal care positively affects the pregnancy process physiologically and psychosocially and reduces adverse birth outcomes such as maternal and infant mortality.^[14]^ In addition, migrants with traumatic backgrounds need emotional and social support, especially during pregnancy.^[15]^ In a meta-analysis, prenatal care was shown to be effective in improving family support and self-care management.^[16]^

Early diagnosis and intervention of depression and anxiety symptoms can help protect and improve the health of migrant mothers and their infants.^[17]^ Turkey is among the countries with the highest number of immigrants in the world. Despite this fact, there are limited studies on depression and anxiety in migrant pregnant women both in Turkey and in other countries. Identification, prevention, treatment and treatment of depression in Syrian immigrant pregnant women and determination of risk factors for its recurrence are of special importance. The aim of this study was to examine the prevalence of depression and anxiety and associated factors in Syrian immigrant pregnant women admitted to Sultanbeyli Empowered Migrant Health Center.

## 2. Methods

This is a cross-sectional study. The population of the study consisted of pregnant women aged 18 to 49 years who applied to Sultanbeyli Empowered Migrant Health Center (GSM) between July 8, 2022 and December 31, 2022. The study was conducted with migrant pregnant women who applied to the Migrant Health Center and agreed to participate in the study. Approved Migrant Health Centers are organizations that provide primary healthcare services to Syrian refugees who have settled in Turkey. These centers are staffed by specialists and general practitioners, dentists, allied health professionals, psychologists and social workers. Most of the staff at these centers are Syrian health workers. Therefore, there is no language barrier. One of the 8 Empowered Migrant Health Centers in İstanbul is located in Sultanbeyli district. Sultanbeyli is the lowest socioeconomic district of İstanbul with a population of 358,201. Approximately 20,000 Syrian asylum seekers live in the district.

### 2.1. Data collection method

For the study, a questionnaire was prepared based on the literature and consisted of 3 sections. The first part of the questionnaire consisted of 42 questions measuring socio-demographic characteristics, risk factors related to current pregnancy and level of knowledge about pregnancy. The Arabic version of the Beck anxiety inventory was included in the second part and the Arabic version of the Beck depression inventory was included in the third part.^[18]^ The questionnaire was conducted as face-to-face interviews with immigrant pregnant women. Interviews with pregnant women were conducted by health workers who spoke Arabic and had received training on the subject beforehand.

### 2.2. Beck depression inventory (BDS)

The BDI was developed to measure the symptoms and level of depression in physical, mental and emotional aspects. The scale is a subjective assessment of how the individual has felt in the last week. The scale is 4-point Likert type and consists of 21 items in total. The options are scored between 0 and 3 and the scores that can be obtained from the scale vary between 0 and 63. The Arabic validity and reliability study of the questionnaire was conducted by Özen and Cerit (2018).^[19]^ In the scores obtained from the scale, 0–9 points indicate that there is no depression symptom, 10 to 16 points indicate mild severe depression, 17 to 29 points indicate moderate severe depression, and 30 to 63 points indicate high severe depression.^[20]^

### 2.3. Beck anxiety inventory

The Beck anxiety inventory consists of 21 questions on a 4-point Likert scale (none, 0; mild, 1; moderate, 2; severe, 3). The score range varies between 0 and 63. BAI is a scale that allows subjective assessment in terms of anxiety symptoms (restlessness, fear, feeling of distress, sweating). The scale assesses how the individual has felt in the last 1 week in terms of anxiety.^[18]^ A validity and reliability study of the Arabic version of the BAI was conducted in Kuwait in 2015.^[21]^ Scores obtained from the scale were evaluated in 3 groups in terms of anxiety; 0–21 points are considered as low risk, 22 to 35 points as medium risk and 36 points and above as high risk.^[22]^

### 2.4. Statistical analysis

For statistical analysis, BDI and BAI were accepted as dependent variables. Descriptive data were presented as number and percentage for categorical variables and mean and standard deviation for continuous variables. Kolmogorov Smirnov and Shapiro–Wilk tests were performed for normality analysis of the data. In significance tests, continuous variables were evaluated with nonparametric tests (Mann–Whitney *U* test for 2 independent groups and Kruskal–Wallis test for more than 2 independent groups) because they did not meet normal distribution conditions. Chi-square and Fisher exact tests were used to compare categorical variables between groups. Statistical significance was accepted as *P* < .05. Statistical analysis of the research data was made with SPSS 24.0 package software.

### 2.5. Permits

Ethics committee approval was obtained from the İstanbul Medi̇pol University’s Non-Invasive Clinical Research Ethics Committee on July 06, 2022 with decision number 603. Informed consent was obtained from all participants after they were informed about the research and permissions.

## 3. Results

The mean age of 274 immigrant pregnant women in the study group was 26.67 ± 5.98 years. Of the participants, 73.0% had primary education and below. A total of 98.9% of the participants are not working. Sociodemographic characteristics of the participants are shown in Table 1.

**Table 1:** Sociodemographic data.

		Mean ± SD	Min–max
Age		26.67 ± 5.98	18–44
Number of people living together		4.97 ± 2.48	1–17
Number of children		1.87 ± 1.45	0–6
Spousal kinship		Number	%
	Yes	83	30.4
	No	190	69.6
Employment status	I work full-time	2	0.7
	I work part-time	1	0.4
	I do not work	263	98.9
Level of education	Below primary education	35	13.0
	Primary education	162	60.0
	High school	59	21.9
	Associate degree/Bachelor’s degree	14	5.1

While 20.4% of pregnant women were pregnant for the first time, 45.5% became pregnant again at least 2 years later. 30.6% of pregnant women had a history of miscarriage. Data on the risky pregnancy status of immigrant pregnant women are shown in Table 2.

**Table 2:** Risky pregnancy status of pregnant women.

		Number (n)	Percentage (%)
First pregnancy status	Yes	56	20.4
	No	218	79.6
At least 2 years have passed since the previous pregnancy	Yes	121	54.5
	No	101	45.5
Birth history over 42 weeks	Yes	11	5.4
	No	211	94.6
Stillborn baby history	Yes	16	7.2
	No	206	92.8
Miscarriage history	Yes	68	30.6
	No	154	69.4
Premature birth history	Yes	6	2.7
	No	216	97.3
Baby with an anomaly	Yes	5	2.3
	No	217	97.7

During pregnancy, 57.2% of the participants did not receive tetanus vaccinations. In the first trimester of pregnancy, 98.9%, 97.8%, and 96.7% knew that they should use iron, vitamin D and folic acid supplements, respectively. Participants knew that 98.9%, 98.50%, 98.5%, and 98.5%, respectively, knew that their child should be exclusively breastfed for the first 6 months, breastfed until at least 2 years of age, and vaccinated. Of the participants, 97.8% knew about postpartum follow-up, 90.5% knew that their child should have a hearing screening test at the hospital, and 89.4% knew that their child’s heel prick blood should be taken after birth. The data measuring the participants’ level of knowledge about pregnancy and motherhood are shown in Table 3.

**Table 3:** Participants’ level of medical knowledge during pregnancy.

		Number (n)	Percentage (%)
*Tetanus vaccination status during pregnancy.*	1 dose	81	29.9
	2 dose	35	12.9
	No	155	57.2
*Do you know that you should take folic acid in the first trimester of pregnancy?*	Yes	262	96.7
	No	9	3.3
*Do you know that you should take vitamin D supplements in the first trimester of pregnancy?*	Yes	267	97.8
	No	6	2.2
*Do you know that you should take iron supplementation in the first trimester of pregnancy?*	Yes	267	97.8
	No	6	2.2
*Do you know that you should have your postpartum follow-up at the family doctor’s office or at the MHC?*	Yes	266	97.8
	No	6	2.2
*Do you know that you should have your child’s heel prick taken after birth?*	Yes	244	89.4
	No	29	10.6
*Do you know that you should have your child’s hearing screening test at the hospital after birth?*	Yes	247	90.5
	No	26	9.5
*Do you know that you should vaccinate your child after birth?*	Yes	269	98.5
	No	4	1.5
*Do you know that your child should be exclusively breastfed for the first 6 months after birth?*	Yes	270	98.9
	No	3	1.1
*After 6 months, do you know that your child should be breastfed with additional food until at least 2 years of age?*	Yes	268	98.5
	No	4	1.5

The majority of participants (99.30%) showed minimal anxiety symptoms. Symptoms of depression were not observed in 97.10% of the participants (Table 4).

**Table 4:** Depression and anxiety status of participants.

Beck anxiety score (mean ± SD)		4.65 ± 3.17	
		Number (n)	Percentage (%)
Anxiety severity n (%)	Minimal (0–21)	272	99.3
	Moderate (22–35)	2	0.7
	Severe (over 36)	0	0
Beck depression score (mean ± SD)		4.48 ± 2.53	
Depression severity n (%)	No symptom severity (0-9)	266	971
	Mildly severe (10–16)	7	2.6
	Moderately severe (17–29)	1	0.4
	Severe (30–63)	0	0.0

84.3% of the participants did not have a chronic disease, while 4% had obesity. 54.4% of pregnant women had never received a COVID-19 vaccine. 69.2% of the participants described their health as very good. None of the participants drank alcohol, while 4% were smokers. 95.7% of the participants had a pregnancy with follow-up, 43.6%, 26.8%, 24.9%, 2.70%, 2.70%, and 1.2% of the participants stated that they had pregnancy follow-up at the Migrant Health Center, Family Medicine, State Hospital, Private Hospital, and Medical Practice, respectively.

The mean Beck’s depression score was found to be 6.55 ± 2.91 in pregnant smokers and 4.4 ± 2.48 in nonsmokers, and the mean Beck’s anxiety score was 6.64 ± 2.25 in pregnant smokers and 4.57 ± 3.19 in nonsmokers. Both anxiety and depression scores were found to be statistically significantly higher in pregnant smokers. The presence of chronic disease, consanguinity, employment status, education level, obesity status, multiple pregnancy, COVID-19, hospitalization, and vaccination status were not statistically significantly different in terms of depression and anxiety. Anxiety and depression and associated factors are shown in Table 5.

**Table 5:** Factors affecting participants’ depression and anxiety.

		n	%	Depression			Anxiety		
				Mean	SD	*P*	Mean	SD	*P*
Chronic disease	Yes	43	15.7	4.44	2.55	.85*	4.42	3.33	.225*
	No	231	84.3	4.49	2.53		4.70	3.15	
Obesity	Yes	11	4.0	4.36	2.80	.799*	4.18	1.25	.992*
	No	261	96.0	4.48	2.53		4.69	3.24	
Status of having COVID-19	Never had	237	86.8	4.42	2.43	.208†	4.66	3.31	.223†
	Once	35	12.8	4.46	2.08		4.46	1.77	
	Twice	1	0.4	19.0	.		12.0		
COVID-19 vaccination status	One dose	47	17.3	4.00	2.40	.3†	4.11	3.07	.296†
	Two doses	70	25.7	4.34	2.09		4.66	2.5	
	Three doses	7	2.6	5.71	1.70		4.86	1.86	
	None	148	54.4	4.62	2.78		4.83	3.55	
How do you rate your health	Moderate	4	1.5	3.75	2.87	.084†	6.75	4.86	.098†
	Good	80	29.3	3.92	3.03		5.66	4.95	
	Very good	189	69.2	4.72	2.26		4.19	1.8	
Spousal kinship	Yes	83	30.4	4.34	2.42	0.613*	4.90	3.67	.42*
	No	190	69.6	4.54	2.59		4.55	2.95	
Smoking	Yes	11	4.0	6.55	2.91	.004*	6.64	2.25	.001*
	No	263	96.0	4.40	2.48		4.57	3.19	
Level of education	Illiterate	32	11.9	4.06	2.15	.644†	3.62	1.56	.45†
	Literate	3	1.1	2.67	3.79		5.00	1.73	
	Primary education	162	60.0	4.43	2.24		4.57	2.52	
	High School	59	21.9	4.93	3.39		5.59	5.11	
	Associate/ Bachelor’s Degree	14	5.2	4.86	1.92		4.21	1.89	
Employment status	I work full-time	2	0.8	5.50	0.71	.343†	3.00	1.41	.296†
	I work part-time	1	0.4	2.00	.		3.00	.	
	I do not work	263	98.9	4.47	2.53		4.68	3.23	
Income level	Under 5.324 TL	252	95.0	4.36	2.36	.152†	4.58	2.91	.286†
	5.324 TL-17.340 TL	12	4.5	5.58	2.43		5.83	6.16	
	Above 17.340 TL	1	0.5	3.00	.		7.00		
Number of people living together at home	5 and less	184	67.2	4.61	2.67	.485*	4.83	3.41	.213*
	Over 5	90	32.8	4.22	2.22		4.30	2.62	
Number of children	3 and less	239	87.2	4.49	2.60	.729*	4.76	3.33	.25*
	4 and more	35	12.8	4.43	2.06		3.97	1.65	
Age	Under 25	103	37.6	4.17	2.53	.102	4.20	1.75	.205†
	Between 25-35	119	43.4	4.71	2.58		4.50	2.47	.205†
	Older than 35	23	8.4	5.09	1.12		3.70	1.82	.205†

TL = Turkish Lira.

* Mann–Whitney U.

† Kruskal–Wallis.

The mean anxiety score was found to be 3.98 ± 2.43 in migrants with first pregnancy and 4.86 ± 3.40 in those with previous pregnancies. Anxiety score was found to be statistically significantly lower in those with a first pregnancy. The mean depression score was found to be 2.33 ± 3.68 in those who had a history of birth over 42 weeks and 4.62 ± 2.48 in those who did not. There is a statistically significant difference between the groups. There was no statistically significant difference between the groups in terms of the effect of the factors of at least 2 years since the previous pregnancy, over 4000 g, under 2500 g, over 42 weeks, preterm birth, stillbirth, miscarriage, having a history of anomalous baby on depression and anxiety scores. Depression and anxiety data are shown in Table 6.

**Table 6:** Factors affecting participants’ depression and anxiety.

Characteristics of previous pregnancies				Depression			Anxiety		
		n	%	Mean	SD	*P*	Mean	SD	*P*
First pregnancy	Yes	56	21.2	4.41	2.51	.409*	3.98	2.43	.008*
	No	208	78.8	4.52	2.54		4.86	3.40	
At least 2 years after the previous pregnancy	Yes	121	54.5	4.64	2.63	.648*	4.88	3.24	.309*
	No	101	45.5	4.37	2.53		4.89	3.59	
Birth history over 4000 g	Yes	11	5.4	5.36	5.08	.942*	4.27	3.44	.379*
	No	191	94.6	4.53	2.41		4.95	3.32	
Birth history under 2500 g	Yes	12	5.4	4.75	3.86	.938*	9.08	7.97	.117*
	No	210	94.6	4.47	2.52		4.66	2.78	
Birth history over 42 weeks	Yes	12	5.4	2.33	3.68	.009*	6.75	7.05	.677*
	No	211	94.6	4.62	2.48		4.77	3.06	
Stillborn baby history	Yes	16	7.2	4.69	2.5	.702*	5.00	2.19	.498*
	No	206	92.8	4.48	2.62		4.87	3.42	
Miscarriage history	Yes	68	30.6	4.87	2.61	.111*	4.34	2.15	.939*
	No	154	69.4	4.31	2.58		5.12	3.81	
Premature birth history	Yes	6	2.7	3.00	1.90	.087*	5.33	3.01	.732*
	No	216	97.3	4.52	2.60		4.87	3.42	
Anomalous baby history	Yes	5	2.3	3.60	2.61	.416*	7.60	8.35	.637*
	No	217	97.7	4.50	2.59		4.82	3.22	

* Mann–Whitney U.

## 4. Discussion

Immigrant pregnant women are a group that should be prioritized in terms of public health practices because they are socially and economically disadvantaged. It is important to identify the mental health problems experienced by immigrant pregnant women. Anxiety and depression are critically important determinants of mental well-being in immigrant pregnant women. In this study, we aimed to determine the anxiety and depression levels of Syrian immigrant pregnant women and to evaluate the associated factors. It was found that 98.5% of the immigrant pregnant women in the study group had good or very good health, 95.0% had low income, 73% had primary education or less, and 12.8% had 4 or more children.

The results of this study provide important information on the age, educational level and socioeconomic status of immigrant pregnant women. The mean age obtained in this study indicates a young population in parallel with similar studies in the literature.^[23]^ It is observed that migrant pregnant women have low-income levels and the majority of them are not employed. This reflects the economic difficulties and unemployment problem faced by the migrant population in general.

In our study, pregnant women under temporary protection who migrated to Turkey were analyzed. The average number of cohabitants of pregnant women was found to be 5, minimum 1 and maximum 17. The maximum number of 17 reveals the difficult living conditions of migrants.

In this population under temporary protection, 30.4% of participants (83 people) were related to their spouse. A meta-analysis of Syrian migrant women in Turkey showed that the rate of consanguineous marriage was 56.0% and the rate of prenatal care was inadequate. It was also shown that the rate of modern family planning use among women in all age groups was 24.0% and unmet need for family planning was 35%.^[24]^ In another meta-analysis, the rate of antenatal care was 64.0%.^[25]^ Although our results are low compared to other studies, this high rate necessitates the importance of genetic screening and investigation of the demographic structure due to immigration.54.4% of pregnant women have never received COVID-19 vaccine before. This shows that pregnant women are hesitant about COVID-19 vaccines.^[26]^ Lack of adequate and accurate information about vaccines and reservations about the side effects of vaccines may have led to low vaccination rates.

Antenatal care is very important in terms of maternal and neonatal morbidity and mortality. Access to health services among migrant women affects antenatal care status and rates.^[27]^ 95.7% of the participants had a followed-up pregnancy. 43.6% of the participants have pregnancy follow-up only at Migrant Health Centers. In addition, 26.8% of the other participants had pregnancy follow-up at Family Medicine, 24.9% at State Hospital, 2.70% at Private Hospital and 1.20% at Medical Practice. Those who had a pregnancy with follow-up generally prefer Migrant Health Centers, Family Medicine and State Hospitals. In similar study, it was found that pregnancy follow-ups were high at the Migrant Health Center.^[26]^ Various studies conducted in Turkey have shown that Syrian immigrant pregnant women have difficulties in accessing prenatal care services and have low rates of prenatal care (73.3%).^[28–30]^ The rate of prenatal care for pregnant women in Turkey is 96.1%.^[31]^ The rate of prenatal care in our research group is very close to the rate in Türkiye. The results of the study show that Syrian pregnant women do not experience problems in accessing health services. This shows the positive effects of government and local authorities’ efforts to facilitate access to health services. In addition, Syrian immigrants’ free access to health services in Türkiye may have contributed to this. It is thought that migrant health centers are highly preferred for follow-ups due to the presence of Syrian healthcare professionals and the absence of language barrier. The importance of language barrier for migrants’ access to healthcare services has been reported in studies.^[32]^

In our study, 57.2% of the participants stated that they did not receive tetanus vaccination during pregnancy, and while 95.7% of the pregnant women continued their follow-up, the fact that 57.2% were not vaccinated may be due to insufficient knowledge about vaccines or hesitation about vaccination. During pregnancy, the majority of pregnant women were aware of the need for iron, vitamin D and folic acid supplementation. It is also known that exclusive breastfeeding for the first 6 months, breast-feeding until at least 2 years of age, immunizations for the child, follow-up of the puerperium after birth, a hearing screening test and heel prick blood collection are necessary. It was found that the health education and counseling provided for pregnant women in the center where the study was conducted provided a positive result at the level of knowledge in pregnant women. In addition, the vast majority of Syrian immigrants in Türkiye (97.7%) reside in cities and 95.1% have been living there for 5 years or more.^[33]^ Living in Türkiye for a long time may have enabled Syrian immigrants to know how the health system works and to use health services effectively.

Having adequate knowledge about vaccines and nutritional supplements during pregnancy is important for migrant women who have difficulties in accessing prenatal care services. Studies have reported that migrant women face various difficulties in accessing prenatal care services and in some cases may encounter misconceptions.^[34]^ Therefore, migrant women should be provided with accurate information and their access to antenatal care services should be facilitated. It is also recommended to conduct awareness raising activities on hearing screening test and heel prick blood collection. In a study conducted in Turkey, attitudes and practices related to breastfeeding were investigated and it was reported that the majority of mothers had accurate knowledge about exclusive breastfeeding for the first 6 months, but there were also false beliefs.^[35]^

It is known that symptoms of depression and anxiety increase during pregnancy and this condition causes adverse obstetric, fetal and neonatal outcomes.^[36]^ The prevalence of anxiety and depression is also known to be higher in migrant pregnant women.^[37]^ The global prevalence of perinatal depression is high (11.9–25.6%) and the clinical anxiety level is 30.5%.^[38,39]^ In addition, the prevalence of anxiety during pregnancy has been found to be higher in the pandemic.^[39]^ In a study conducted in Malaysia, anxiety was found to be 9.1% and depression 8% in antenatal mothers.^[40]^ In a study conducted with immigrant women in Taiwan, it was shown that depression scores increased in women from pregnancy to the 3rd month postpartum.^[41]^ Various studies conducted with pregnant women in Turkey have shown that 27.9% to 30.9% of them experience symptoms of depression at a level that requires treatment.^[42,43]^ Several studies have shown that anxiety and depression in migrant pregnant women are associated with low education and low-income level,^[38,44]^ lack of social support,^[43]^ past traumatic physical and psychological experiences^[37,43,45]^ and history of miscarriage.^[44]^ In a meta-analysis conducted during the COVID-19 pandemic and involving 15,050 pregnant women, it was shown that reduced perception of general support, low household socioeconomic status, and prepregnancy chronic conditions increased the risk of anxiety.^[34]^ In our study, 4% of the participants were smokers. Similarly, both anxiety and depression scores were found to be statistically significantly higher in pregnant smokers. It is also known that limitation of walking and physical activity can affect dysphoric mood and anxiety in late pregnancy.^[46]^

Our study was conducted during the pandemic period. Minimal anxiety was detected in 99.3% of the participating Syrian immigrant pregnant women. In addition, no depressive symptoms were detected in 97.1% of the immigrant pregnant women. Anxiety and depression levels in our study are very low compared to the literature. In addition, in our study, 93.30% of the immigrant pregnant women had a low income level (˂5324 Turkish Lira), 73.0% had a low education level (primary education and below) and 15.7% had chronic diseases. Syrian migrants living in Türkiye culturally lead a multi-population family life. However, their low socioeconomic status stands out as a challenging factor in crowded family life. These negative socioeconomic factors may have contributed positively to the development of the social support mechanism within the family. On the other hand, Syrian immigrants’ efforts to reside in cities at a distance where they can communicate and interact with each other support them to maintain social relations at the social level. This may have contributed to the lower incidence of anxiety and depression in immigrant pregnant women. In addition, it is thought that the social support programs carried out by public institutions and nongovernmental organizations in Türkiye for immigrants have an effect on the low incidence of anxiety and depression.

Perceived social support was found to be a statistically significant factor affecting both depression and anxiety experience.^[42]^ Low adaptation to the host culture was found to be associated with high maternal perinatal depression. Strategies to help immigrant women resolve possible cultural conflicts should be developed to reduce their depression.^[41]^ A study involving 3894 pregnant women found that online psychological interventions significantly reduced depressive and anxiety symptoms after the intervention and that psychological interventions adopting a cognitive behavioral therapy or mindfulness therapy component showed beneficial effects for improving depressive symptoms among pregnant women.^[47]^ Another study reported that an unplanned and possibly unwanted pregnancy caused emotional distress.^[48]^ In our study, the mean number of children of pregnant women was found to be 1.87 ± 1.45 with a minimum of 1 and a maximum of 6. In order to prevent unplanned pregnancies in the immigrant population with insufficient socioeconomic status, it is important to provide family planning materials, to deliver them to people and to inform them.

### 4.1. Strengths and limitations

Our study was conducted in a large population of pregnant women, a vulnerable group in Turkey with a large immigrant population. The language barrier was eliminated by using an Arabic questionnaire.

On the other hand, since the questionnaires were conducted face-to-face, data losses are also observed due to missing answers in some questions. Since health center-based data is collected, there may be limitations in terms of reflecting the society and generalizability.

## 5. Conclusion

Anxiety and depression levels were found to be minimal in immigrant pregnant women in our research group. Minimal anxiety was detected in 99.3% of Syrian immigrant pregnant women. No depressive symptoms were observed in 97.1% of the participants. Both anxiety and depression scores were found to be statistically significantly higher in pregnant smokers. Establishing and sustaining interventions and support programs for the psychosocial health of immigrant pregnant women will help both mothers and babies to have a healthy pregnancy. Such programs can alleviate difficulties during pregnancy and improve the quality of life of immigrant women by providing services to overcome factors such as loneliness, language problems and lack of social support. It would be useful to conduct comprehensive research on the target group to determine the factors that enable Syrian immigrant pregnant women in Türkiye to have low levels of anxiety and depression and to evaluate and sustain the impact of these factors. Assessing the prevalence and associated factors of depression and anxiety in immigrant pregnant women is important for developing services and supports to address the needs of these women. In addition, facilitating immigrant pregnant women’s access to health services and training health care professionals in working with immigrant women is an important part of an effective intervention and prevention strategy for depression and anxiety disorders in this population. It is important to evaluate all health and social support services for immigrant pregnant women, especially preventive health services, in terms of access and utilization, and to carry out studies to ensure that they are structured to cover all immigrant pregnant women. In this way, all immigrant pregnant women will have easy access to the services they need.

## Author contributions

**Conceptualization:** Muhammed Atak, Mehmet Akif Sezerol.

**Data curation:** Muhammed Atak, Mehmet Akif Sezerol.

**Formal analysis:** Muhammed Atak, Mehmet Akif Sezerol, Elif Nur Koçak, Mehmet Sait Değer.

**Funding acquisition:** Muhammed Atak, Mehmet Akif Sezerol.

**Investigation:** Muhammed Atak, Mehmet Akif Sezerol, Mehmet Sait Değer.

**Methodology:** Muhammed Atak, Mehmet Akif Sezerol, Mehmet Sait Değer

**Project administration:** Muhammed Atak, Mehmet Akif Sezerol.

**Resources:** Muhammed Atak, Mehmet Akif Sezerol, Elif Nur Koçak, Mehmet Sait Değer, Hamza Kurubal.

**Software:** Muhammed Atak, Mehmet Akif Sezerol, Elif Nur Koçak, Mehmet Sait Değer, Hamza Kurubal.

**Supervision:** Muhammed Atak, Mehmet Akif Sezerol.

**Validation:** Muhammed Atak, Mehmet Akif Sezerol, Elif Nur Koçak, Hamza Kurubal.

**Visualization:** Muhammed Atak, Mehmet Akif Sezerol, Elif Nur Koçak, Hamza Kurubal.

**Writing – original draft:** Muhammed Atak, Mehmet Akif Sezerol, Elif Nur Koçak, Mehmet Sait Değer, Hamza Kurubal.

**Writing – review & editing:** Muhammed Atak, Mehmet Akif Sezerol, Elif Nur Koçak, Mehmet Sait Değer, Hamza Kurubal.
